# Supplementary material for: Psychometric evaluation of a quality of recovery score for the postanesthesia care unit—A preliminary validation study
Source: PLoS One. 2023 Aug 15;18(8):e0289685. doi: 10.1371/journal.pone.0289685 (PMC10426991; doi:10.1371/journal.pone.0289685)
Supplement: S2 Table — (DOCX) [file pone.0289685.s002.docx]

| ***Waren Sie seit Ihrer Ankunft im Aufwachraum von diesen Beschwerden betroffen?*** | | | | | |
| --- | --- | --- | --- | --- | --- |
|  | 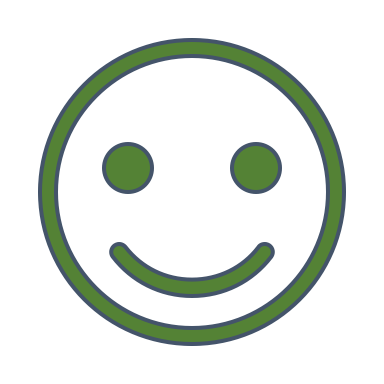  nie | 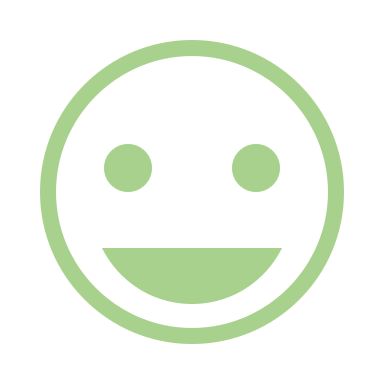  selten | 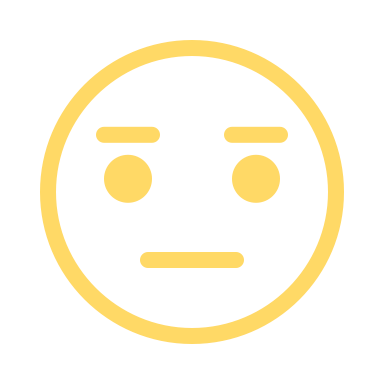  gelegentlich | 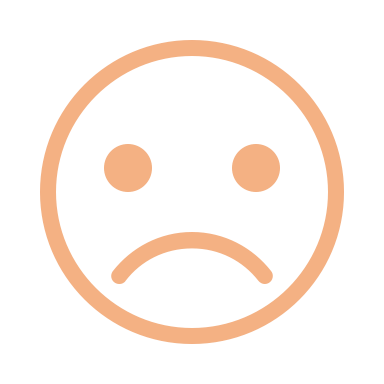  meistens | 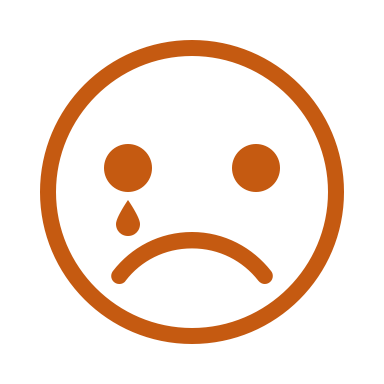  immer |
| 1. Schmerzen |  |  |  |  |  |
| 2. Heiserkeit |  |  |  |  |  |
| 3. Trockener Mund |  |  |  |  |  |
| 4. Atembeschwerden |  |  |  |  |  |
| 5. Übelkeit |  |  |  |  |  |
| 6. Erbrechen |  |  |  |  |  |
| 7. Frieren |  |  |  |  |  |
| 8. Benommenheit |  |  |  |  |  |
| ***Waren seit Ihrer Ankunft im Aufwachraum von diesen Beschwerden betroffen?*** | | | | | |
|  | 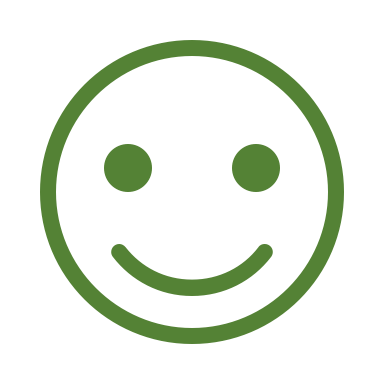  nie | 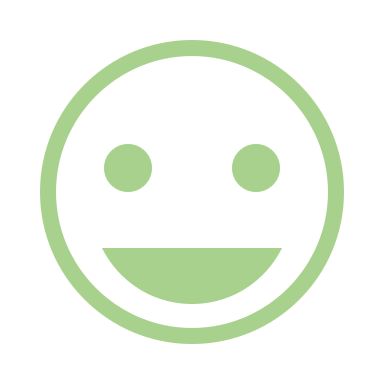  selten | 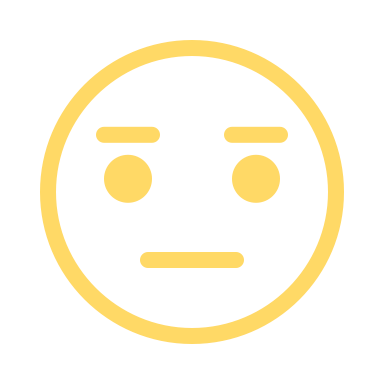  gelegentlich | 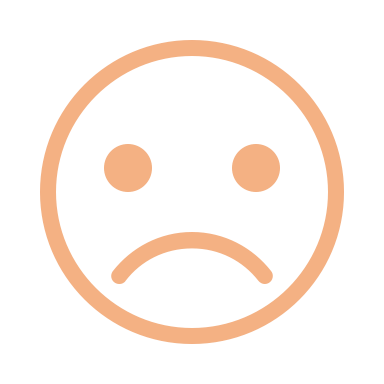  meistens | 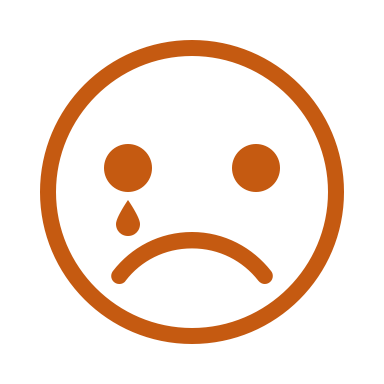  immer |
| 9. Verwirrtheit |  |  |  |  |  |
| 10. Ängstlichkeit |  |  |  |  |  |
| ***Wie würden Sie Ihr Befinden seit Ihrer Ankunft im Aufwachraum beschreiben?*** | | | | | |
|  | 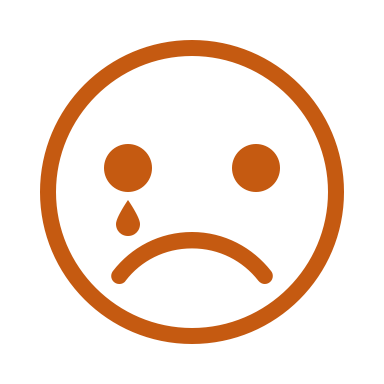  nie | 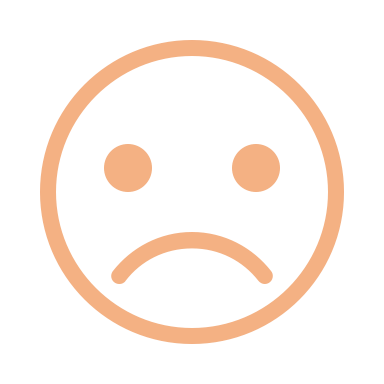  selten | 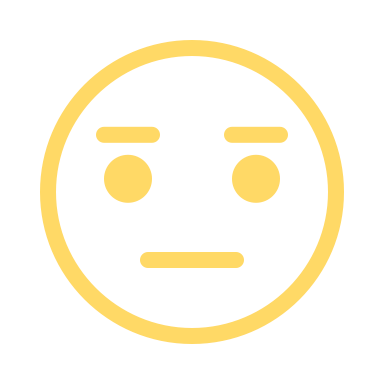  gelegentlich | 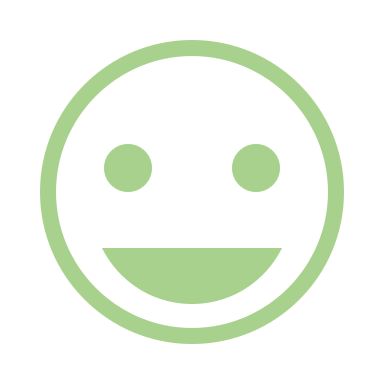  meistens | 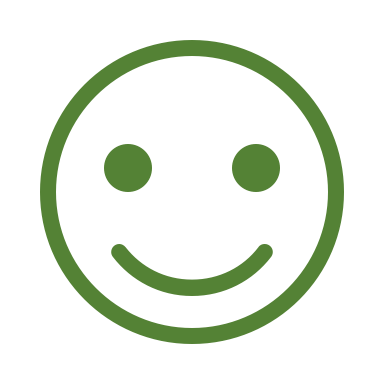  immer |
| 11. Ich verstehe Anweisungen und Ratschläge. |  |  |  |  |  |
| 12. Ich fühle mich den Umständen  entsprechend wohl. |  |  |  |  |  |
| 13. Ich fühle mich gut durch ärztliches und Pflegepersonal betreut. |  |  |  |  |  |
