# Supplementary material for: Psychometric evaluation of a quality of recovery score for the postanesthesia care unit—A preliminary validation study
Source: PLoS One. 2023 Aug 15;18(8):e0289685. doi: 10.1371/journal.pone.0289685 (PMC10426991; doi:10.1371/journal.pone.0289685)
Supplement: S3 Table — Postoperative assessment: Inter-item correlations for the 13 items of the QoR-PACU score. Correlations are expressed as Pearson correlation coefficients. (DOCX) [file pone.0289685.s003.docx]

**S3 Table. Postoperative interitem correlation.**

| **QoR-PACU item** | **1** | **2** | **3** | **4** | **5** | **6** | **7** | **8** | **9** | **10** | **11** | **12** | **13** |
| --- | --- | --- | --- | --- | --- | --- | --- | --- | --- | --- | --- | --- | --- |
| **1** | --- |  |  |  |  |  |  |  |  |  |  |  |  |
| **2** | 0.202 | --- |  |  |  |  |  |  |  |  |  |  |  |
| **3** | 0.280 | 0.427 | --- |  |  |  |  |  |  |  |  |  |  |
| **4** | 0.147 | 0.130 | 0.196 | --- |  |  |  |  |  |  |  |  |  |
| **5** | 0.115 | 0.109 | 0.123 | 0.259 | --- |  |  |  |  |  |  |  |  |
| **6** | 0.074 | -0.032 | 0.041 | 0.072 | 0.398 | --- |  |  |  |  |  |  |  |
| **7** | 0.083 | 0.031 | 0.036 | 0.046 | 0.049 | -0.023 | --- |  |  |  |  |  |  |
| **8** | 0.087 | 0.177 | 0.240 | 0.036 | 0.283 | 0.103 | 0.141 | --- |  |  |  |  |  |
| **9** | 0.044 | 0.062 | 0.061 | 0.046 | 0.107 | 0.037 | 0.147 | 0.337 | --- |  |  |  |  |
| **10** | 0.125 | -0.008 | 0.180 | 0.216 | 0.130 | 0.120 | 0.039 | 0.096 | 0.270 | --- |  |  |  |
| **11** | -0.032 | 0.016 | 0.062 | 0.168 | 0.172 | 0.115 | 0.060 | 0.212 | 0.449 | 0.298 | --- |  |  |
| **12** | 0.222 | 0.055 | 0.227 | 0.198 | 0.274 | 0.140 | 0.121 | 0.273 | 0.197 | 0.342 | 0.406 | --- |  |
| **13** | 0.053 | 0.106 | 0.087 | 0.134 | 0.127 | 0.077 | 0.051 | 0.058 | 0.229 | 0.331 | 0.356 | 0.341 | --- |

**S3 Table**: Postoperative assessment: Interitem correlations for the 13 items of the QoR-PACU score. Correlations are expressed as Pearson correlation coefficients.
