# Supplementary material for: Psychometric evaluation of a quality of recovery score for the postanesthesia care unit—A preliminary validation study
Source: PLoS One. 2023 Aug 15;18(8):e0289685. doi: 10.1371/journal.pone.0289685 (PMC10426991; doi:10.1371/journal.pone.0289685)
Supplement: Attachment — Submitted filename: PONE-D-22-18272_reviewer.pdf [file pone.0289685.s004.pdf]

PLOS ONE

Psychometric evaluation of a quality of recovery score for the postanesthesia care unit – a prospective validation study

--Manuscript Draft--

**Manuscript Number:**	PONE-D-22-18272
**Article Type:**	Research Article
**Full Title:**	Psychometric evaluation of a quality of recovery score for the postanesthesia care unit – a prospective validation study
**Short Title:**	Development of a quality of recovery score for the postanesthesia care unit
**Corresponding Author:**	Marlene Fischer, M.D., Ph.D.<br>University Medical Center Hamburg-Eppendorf: Universitatsklinikum Hamburg-Eppendorf<br>Hamburg, GERMANY
**Keywords:**	Quality of recovery; postanesthesia care unit; patient-reported outcome; recovery from anesthesia; perioperative care
**Abstract:**	Introduction<br><br>Patients' perception of postoperative recovery is a key aspect of perioperative care. Self-reported quality of recovery (QoR) has evolved as a relevant endpoint in perioperative research. Several psychometric instruments have been introduced to assess self-reported recovery 24 hours after surgery. However, there is no questionnaire suitable for use in the postanesthesia care unit (PACU). We aimed to develop and psychometrically evaluate a QoR questionnaire for the PACU (QoR-PACU).<br><br>Methods<br><br>The QoR-PACU was developed based on the 40-item QoR-40 questionnaire. Adult patients scheduled for elective urologic surgery completed the QoR-PACU preoperatively and during the PACU stay. We evaluated feasibility, validity, reliability, and responsiveness. Between March and November 2020 we enrolled 375 patients. After a pretest phase the questionnaire was modified twice to ensure ease of understanding.<br><br>Results<br><br>We administered the final version of the QoR-PACU to 255 patients, with a completion rate of 96.5%. Construct validity was good with postoperative QoR-PACU sum scores correlating with age (r= 0.23, 95%CI: 0.11 to 0.35, p <0.001), length of PACU stay (r= -0.15, 95%CI: -0.27 to -0.03, p= 0.02), pain in the PACU (r= -0.48, 95%CI: -0.57 to -0.37, p <0.001) and piritramide dose administered (r= -0.29, 95%CI: -0.40 to -0.17, p <0.001). Cronbach's alpha was 0.67 (95%CI: 0.61 – 0.73) with moderate test-retest reliability (ICC of 0.67, 95%CI: 0.38 – 0.83). Cohen's effect size was 3.08 and the standardized response mean was 1.65 indicating adequate responsiveness.<br><br>Conclusion<br><br>The assessment of QoR in the early postoperative period is feasible. We found high acceptability with excellent recruitment and successful completion rates, adequate responsiveness, and sufficent validity and reliability. Future studies should evaluate the psychometric properties of the QoR-PACU in more heterogeneous patient populations including female and gender-diverse patients with varying degress of perioperative risk.
**Order of Authors:**	Ursula Kahl, M.D.
	Katrin Brodersen, M.D.
	Sarah Kaiser

Powered by Editorial Manager® and ProduXion Manager® from Aries Systems Corporation

	Linda Krause, M.Sc.
	Regine Klinger, PhD
	Lili Plümer, M.D.
	Christian Zöllner, M.D.
	Marlene Fischer, M.D., PhD
**Additional Information:**	
**Question**	**Response**
**Financial Disclosure**<br><br>Enter a financial disclosure statement that describes the sources of funding for the work included in this submission. Review the submission guidelines for detailed requirements. View published research articles from *PLOS ONE* for specific examples.<br><br>This statement is required for submission and **will appear in the published article** if the submission is accepted. Please make sure it is accurate.<br><br>**Unfunded studies**<br>Enter: *The author(s) received no specific funding for this work.*<br><br>**Funded studies**<br>Enter a statement with the following details:<br>• Initials of the authors who received each award<br>• Grant numbers awarded to each author<br>• The full name of each funder<br>• URL of each funder website<br>• Did the sponsors or funders play any role in the study design, data collection and analysis, decision to publish, or preparation of the manuscript?<br>• **NO** - Include this sentence at the end of your statement: *The funders had no role in study design, data collection and analysis, decision to publish, or preparation of the manuscript.*<br>• **YES** - Specify the role(s) played.<br><br>* typeset	Ursula Kahl was funded by the Clinician Scientist Program of the medical faculty of the University of Hamburg, during the conduct of the study. The University of Hamburg was not involved in any of the following: study design, conduct of the research, preparation of this manuscript, analysis and interpretation of data; writing of the report; decision to submit the article for publication.
**Competing Interests**<br><br>Use the instructions below to enter a competing interest statement for this submission. On behalf of all authors,	The authors have declared that no competing interests exist.

disclose any competing interests that could be perceived to bias this work—acknowledging all financial support and any other relevant financial or non-financial competing interests.<br><br>This statement is required for submission and **will appear in the published article** if the submission is accepted. Please make sure it is accurate and that any funding sources listed in your Funding Information later in the submission form are also declared in your Financial Disclosure statement.<br><br>View published research articles from *PLOS ONE* for specific examples.<br><br>**NO authors have competing interests**<br><br>Enter: *The authors have declared that no competing interests exist.*<br><br>**Authors with competing interests**<br><br>Enter competing interest details beginning with this statement:<br><br>*I have read the journal's policy and the authors of this manuscript have the following competing interests: [insert competing interests here]*<br><br>* typeset	
**Ethics Statement**<br><br>Enter an ethics statement for this submission. This statement is required if the study involved:<br><br>• Human participants<br>• Human specimens or tissue<br>• Vertebrate animals or cephalopods<br>• Vertebrate embryos or tissues<br>• Field research<br><br>Write "N/A" if the submission does not require an ethics statement.	Ethical approval was obtained from the ethics committee at the Hamburg State Chamber of Physicians on February 11, 2020 (serial number PV7218). Each patient gave written informed consent before the initiation of study-related procedures.

General guidance is provided below. Consult the submission guidelines for detailed instructions. **Make sure that all information entered here is included in the Methods section of the manuscript.**<br><br>**Format for specific study types**<br><br>**Human Subject Research (involving human participants and/or tissue)**<br>• Give the name of the institutional review board or ethics committee that approved the study<br>• Include the approval number and/or a statement indicating approval of this research<br>• Indicate the form of consent obtained (written/oral) or the reason that consent was not obtained (e.g. the data were analyzed anonymously)<br><br>**Animal Research (involving vertebrate animals, embryos or tissues)**<br>• Provide the name of the Institutional Animal Care and Use Committee (IACUC) or other relevant ethics board that reviewed the study protocol, and indicate whether they approved this research or granted a formal waiver of ethical approval<br>• Include an approval number if one was obtained<br>• If the study involved *non-human primates*, add *additional details* about animal welfare and steps taken to ameliorate suffering<br>• If anesthesia, euthanasia, or any kind of animal sacrifice is part of the study, include briefly which substances and/or methods were applied<br><br>**Field Research**<br><br>Include the following details if this study involves the collection of plant, animal, or other materials from a natural setting:<br>• Field permit number<br>• Name of the institution or relevant body that granted permission	
**Data Availability**<br><br>Authors are required to make all data	No - some restrictions will apply

Powered by Editorial Manager® and ProduXion Manager® from Aries Systems Corporation

underlying the findings described fully available, without restriction, and from the time of publication. PLOS allows rare exceptions to address legal and ethical concerns. See the PLOS Data Policy and FAQ for detailed information.<br><br>A Data Availability Statement describing where the data can be found is required at submission. Your answers to this question constitute the Data Availability Statement and **will be published in the article**, if accepted.<br><br>**Important:** Stating 'data available on request from the author' is not sufficient. If your data are only available upon request, select 'No' for the first question and explain your exceptional situation in the text box.<br><br>Do the authors confirm that all data underlying the findings described in their manuscript are fully available without restriction?	
**Describe where the data may be found in full sentences. If you are copying our sample text, replace any instances of XXX with the appropriate details.**<br><br>• If the data are **held or will be held in a public repository**, include URLs, accession numbers or DOIs. If this information will only be available after acceptance, indicate this by ticking the box below. For example: *All XXX files are available from the XXX database (accession number(s) XXX, XXX.).*<br>• If the data are all contained **within the manuscript and/or Supporting Information files**, enter the following: *All relevant data are within the manuscript and its Supporting Information files.*<br>• If neither of these applies but you are able to provide **details of access elsewhere**, with or without limitations, please do so. For example:<br><br>*Data cannot be shared publicly because of [XXX]. Data are available from the*	Data cannot be shared publicly because of the German General Data Protection Regulation. Data are available from the corresponding author Marlene Fischer (contact via mar.fischer@uke.de) for researchers who meet the criteria for access to confidential data.

XXX Institutional Data Access / Ethics Committee (contact via XXX) for researchers who meet the criteria for access to confidential data.<br><br>*The data underlying the results presented in the study are available from (include the name of the third party and contact information or URL).*<br>• This text is appropriate if the data are owned by a third party and authors do not have permission to share the data.<br><br>* typeset	
Additional data availability information:	

Powered by Editorial Manager® and ProduXion Manager® from Aries Systems Corporation

Manuscript

Click here to access/download;Manuscript;PACQoR3_manuscript_300622.do

Title

Psychometric evaluation of a quality of recovery score for the postanesthesia care unit – a prospective validation study

Authors

Ursula Kahl[1], Katrin Brodersen[1], Sarah Kaiser[1], Linda Krause[2], Regine Klinger[1], Lili Plümer[1], Christian Zöllner[1], Marlene Fischer[1,3]

Affiliations

[1]Department of Anesthesiology, University Medical Center Hamburg-Eppendorf, Hamburg, Germany;

[2]Institute of Medical Biometry and Epidemiology, University Medical Center Hamburg-Eppendorf, Hamburg, Germany;

[3]Department of Intensive Care Medicine, University Medical Center Hamburg-Eppendorf, Hamburg, Germany.

***Corresponding author**

Marlene Fischer, MD, Ph

Department of Anaesthesiology,

University Medical Center Hamburg-Eppendorf

Martinistrasse 52

20246 Hamburg, Germany

Phone: +4915222827500

Email: mar.fischer@uke.de

Details of authors' contributions

UK acquisition, analysis and interpretation of data, drafting the article

KB acquisition, analysis and interpretation of data, drafting the article

SK acquisition, substantial revision of the manuscript

LK analysis and interpretation of data, substantial revision of the manuscript

RK interpretation of data, substantial revision of the manuscript

CZ conception and design of the work, substantial revision of the manuscript

MF conception and design of the work, acquisition, analysis and interpretation of data, drafting the article

Abstract (max. 300 words)

Introduction: Patients' perception of postoperative recovery is a key aspect of perioperative care. Self-reported quality of recovery (QoR) has evolved as a relevant endpoint in perioperative research. Several psychometric instruments have been introduced to assess self-reported recovery 24 hours after surgery. However, there is no questionnaire suitable for use in the postanesthesia care unit (PACU). We aimed to develop and psychometrically evaluate a QoR questionnaire for the PACU (QoR-PACU).

Methods: The QoR-PACU was developed based on the 40-item QoR-40 questionnaire. Adult patients scheduled for elective urologic surgery completed the QoR-PACU preoperatively and during the PACU stay. We evaluated feasibility, validity, reliability, and responsiveness. Between March and November 2020 we enrolled 375 patients. After a pretest phase the questionnaire was modified twice to ensure ease of understanding.

Results: We administered the final version of the QoR-PACU to 255 patients, with a completion rate of 96.5%. Construct validity was good with postoperative QoR-PACU sum scores correlating with age (r= 0.23, 95%CI: 0.11 to 0.35, p <0.001), length of PACU stay (r= -0.15, 95%CI: -0.27 to -0.03, p= 0.02), pain in the PACU (r= -0.48, 95%CI: -0.57 to -0.37, p <0.001) and piritramide dose administered (r= -0.29, 95%CI: -0.40 to -0.17, p <0.001). Cronbach's alpha was 0.67 (95%CI: 0.61 – 0.73) with moderate test-retest reliability (ICC of 0.67, 95%CI: 0.38 – 0.83). Cohen's effect size was 3.08 and the standardized response mean was 1.65 indicating adequate responsiveness.

Conclusion: The assessment of QoR in the early postoperative period is feasible. We found high acceptability with excellent recruitment and successful completion rates, adequate responsiveness, and sufficent validity and reliability. Future studies should evaluate the

psychometric properties of the QoR-PACU in more heterogeneous patient populations including female and gender-diverse patients with varying degress of perioperative risk.

Key words

Quality of recovery, postanesthesia care unit, patient-reported outcome, recovery from anesthesia, perioperative care.

Introduction

The improvement of postoperative recovery is a common aim of all disciplines involved in perioperative care (1–3). Postoperative recovery after surgery and anesthesia has traditionally been assessed using objective parameters including but not limited to cardiovascular, pulmonary or infectious complications, pain or length of hospital stay (4–6). In recent years patient's perception of recovery after surgery has been increasingly recognized as a relevant outcome measure (1,2,7). To allow for comparability across clinical studies, the *Standardized Endpoints in Perioperative Medicine (StEP)* initiative recommends six standardized outcome measures reflecting patient comfort: postoperative pain, nausea, time to gastrointestinal recovery, time to mobilization, sleep disturbance, and the assessment of postoperative quality of recovery (QoR) (8,9). In the same line, the introduction of patient-reported outcome assessments is recommended by the *American Society for Enhanced Recovery and Perioperative Quality Initiative* (10). Various instruments have been developed to evaluate postoperative patient-reported recovery. Myles and colleagues developed the 40-item QoR-40 questionnaire that has been validated, translated, and used extensively (2,11–14). In 2013, the same research group developed the 15-item QoR-15 questionnaire which is a shorter version of the more extensive QoR-40 (7,15–24). Both instruments have been introduced to assess QoR one day after surgery (2,7). The importance of advanced recovery room care and the assessment of patient-centered outcomes early after surgery has recently been highlighted by an Australian feasibility study (25). Yet, there is no instrument appropriate for application in the postanesthesia care unit (PACU).

The aim of this study was to develop a QoR questionnaire for the PACU (QoR-PACU) and to evaluate its feasibility, validity, reliability, responsiveness, and clinical acceptability in patients after general anesthesia for elective non-cardiac surgery.

Materials and Methods

Ethical approval and study registration

Ethical approval was obtained from the ethics committee at the Hamburg State Chamber of Physicians on February 11, 2020 (serial number PV7218). Each patient gave written informed consent before the initiation of study-related procedures. The study was registered at clinicaltrials.gov (NCT04528537).

Study design and participants

We performed a prospective observational cohort study at the Department of Anesthesiology of a quarternary care university hospital in Northern Germany. We performed a pre-test of the QoR-PACU in a randomly selected cohort of 10 patients to assess feasibility. Subsequenty, all study participants completed the QoR-PACU on the day before surgery to obtain baseline values. Patients were assessed postoperatively 120 minutes after arrival in the PACU allowing for a tolerance interval of ± 60 minutes. A subgroup of patients underwent a second postoperative assessment after another 60 minutes ± 30 to evaluate test-rest reliability. Additionally, test-retest reliability was assessed in a subset of patients on the first postoperative day. All assessments were performed by three examiners (KB, SK, MF).

Patients were included, if they were 18 years or older and received general anesthesia for elective radical prostatectomy. We excluded patients scheduled for Same-Day-Surgery, ambulatory surgery or postoperative admission to the intensive care unit and patients without excellent German language skills.

Development and adaptation of the QoR-PACU

With the permission of Professor Paul Myles, we aimed to develop a questionnaire derived from the QoR-40 to assess the QoR in PACU. Three experienced anesthesiologists (MF, LN, CZ) independently selected 15 items each from the QoR-40, which they deemed to be of high clinical

importance for recovery and self-perceived health status during the early postoperative period. After thorough discussion a consensus version containing 16 items was developed (version 1). Similar to the QoR-15 questionnaire, an 11-point numerical rating scale was used with a score from 0 (“none of the time”) to 10 (“all of the time”). For negative items the scoring was reversed. A total score was calculated ranging from 0 to 160 points, with a higher score representing better recovery. After a successful pretest phase, we administered the 16-item QoR-PACU (version 1) to 72 patients. During the early study period we repeatedly noticed misunderstandings. One major issue was the 11-point response scale from 0 to 10, reflecting the frequency of positive or negative symptoms. A relevant number of patients was confused with simultaneous pain ratings, which are part of clinical practice in the PACU, and assessed intensity rather than frequency. Therefore, we reduced the 11-point scale to a 5-point scale from 0 to 4 (version 2). For ease of understanding we linked each number with an adverb of frequency: 4 points = always, 3 points = most of the time, 2 points = occasionally, 1 point = rarely, 0 points = never, resulting in a total score from 0 to 52 points. For negative items the scoring was reversed. After assessment of another 48 patients, the QoR-PACU questionnaire was reduced to 13 items (version 3, S1 Table, S2 Table). Four items were dropped for lack of importance as reported by patients and PACU staff: the distinction between severe and moderate pain, shivering, bad dreams and the feeling of being alone. The item “nausea and vomiting” was separated into two items.

Data collection

Medical history and demographic characteristics were collected during the preanesthesia visit. We recorded the following clinical data: age, gender, body mass index, Charlson Comorbidity Index (CCI), obstructive sleep apnea syndrome, medication, American Society of Anesthesiologists (ASA) physical status classification, education, and current profession. To preoperatively assess the risk for obstructive sleep apnea syndrome we used the STOP-Bang score that evaluates snoring, tiredness, observed apnea, high blood pressure, body mass index, age, neck

circumference, and male gender with higher scores indicating a higher risk. We retrieved information about the duration of surgery, length of PACU stay, intra- and perioperative medication from anesthesia protocols. The Numeric Rating Scale (NRS) was used to assess pain intensity in the PACU.

Sample Size

There is no consistent recommendation regarding sample size for the development and the evaluation of a questionnaire. A "rule of thumb" suggests at least 10 participants for each scale item (26,27). Others propose a sample size 300 after initial pretesting (28). To meet both criteria we opted for 375 patients including an estimated drop-out rate of 25%.

Statistical analysis

Continuous variables are presented as median with 25th and 75th percentiles. Categorical variables are given as absolute and relative numbers.

Validity was assessed using the postoperative QoR-PACU sum score. To assess construct validity we compared postoperative QoR-PACU sum scores between categories of clinically relevant variables using a linear model and analysis of variance. Additionally, we analyzed correlations between postoperative QoR-PACU sum scores and clinically relevant continuous variables using Pearson's product-moment correlation coefficients and corresponding 95% confidence intervals.

Reliability was analyzed using results of individual items of the postoperative QoR-PACU and sum scores of postoperative QoR-PACU of those who took the tests twice postoperatively. Reliability was assessed based on internal consistency using Cronbach's alpha, split-half reliability and test-retest reliability using Pearson's correlation coefficient. Pearson correlation was chosen over the intraclass correlation coefficient since it is reasonable to assume that the state of the patient changed within the one hour of time between both assessments (29).

Cronbach's alpha was calculated between items of the postoperative QoR-PACU using the alpha function from the R-package "psych" version 2.1.9 (30). An alpha coefficient of 0.70 and higher is considered to be an acceptable threshold for reliability (26). To obtain split-half reliability the function splithalf.r from the "multicon" R-package in the version 1.6 was used on the items of the postoperative QoR-PACU results (31).

Responsiveness refers to the ability to detect clinical important change. Responsiveness was analyzed taking into account pre- and postoperative QoR-PACU sum scores and was expressed with Cohen's effect size and standardized response mean. Cohen's effect size is defined as mean difference between preoperative and postoperative QoR-PACU sum scores divided by the SD of the preoperative QoR-PACU sum scores. Standardized response mean was calculated as the mean difference between pre- and postoperative QoR-PACU sum scores divided by the SD of these differences.

The proportion of patients who successfully completed the QoR-PACU postoperatively was used to assess acceptability and feasibility.

All analyses were done on complete available cases so no imputation of missing data was performed. P-values are presented as descriptive summary measures and do not represent results of confirmatory testing. No adjustment for multiplicity was performed. All analyses were performed with R Statistical Software, version 3.5.3 (R Foundation for Statistical Computing, Vienna, Austria).

Results

Demographic and clinical characteristics

Between March and November 2020, [illegible] patients were approached by the study team for the assessment with version 3. A total of 246 patients completed the final version of the QoR-PACU questionnaire resulting in a completion rate of 96.5%. Figure 1 shows the flow of participants during the course of the study. Details on baseline demographic and clinical characteristics and perioperative variables related to surgery and anesthesia are presented in Table 1.

Figure 1: Flow chart

Fig. 1: The flow diagram shows patients included and excluded throughout the course of the study.

Table 1 : Demographic and clinical characteristics

		n = 246
**Patient characteristics**		
Age (years)		64 (60; 69)
Body Mass Index (kg/m^2)		26.5 (24.5; 28.9)
ASA physical status		
	II	214 (87)
	III	32 (13)
Charlson Comorbidity Index		4 (4; 5)
Obstructive sleep apnea syndrome		17 (7.6)
Education		
	<9 years	2 (0.8)
	9 - 10 years	28 (11.4)
	10 - 12 years	95 (38.6)
	12 - 13 years	16 (6.5)
	University degree	105 (42.7)
**Surgery**		
Duration of surgery (min)		153.0 (135.0; 175.8)
Surgical approach		
	Open retropubic radical prostatectomy	104 (42.3)
	Robot-assisted radical prostatectomy	142 (57.7)

**Anaesthesia and perioperative medication**	
Duration of anaesthesia (min)	223.5 (206.0; 247.8)
Premedication with Midazolam	8 (3.3)
Prophylaxis for postoperative nausea and vomiting	
None	2 (0.8)
Dexamethasone	6 (2.4)
Ondansetron	3 (1.2)
Dexamethasone and Ondansetron	235 (95.5)
Anaesthesia maintenance	
Sevoflurane	241 (98.0)
Propofol	5 (2.0)
Sufentanil (cumulative; µg)	85.0 (70.0; 95.0)
Norepinehphrine (maximum dosage; µg/kg/min)	0.1 (0.07; 0.14)
Fluids	
Crystalloids (ml)	2500 (2000; 3000)
Colloids (ml)	0 (0;0)
**Postoperative care and medication**	
Length of PACU stay (min)	152.0 (118.3;196.5)
Piritramide (cumulative; mg)	3.75 (3.75;7.5)
Pethidine (cumulative; mg)	25.0 (25.0;25.0)
Discharge to	
Normal ward	208 (84.6)
Scheduled overnight PACU stay	27 (11.0)
Unscheduled overnight PACU stay	11 (4.5)

Table 1: Demographic and clinical characteristics. Data are presented as median (25th; 75th percentile) or n (%). ASA: American Society of Anesthesiologists. PACU: postanaesthesia care unit.

QoR-PACU

Median QoR-PACU sum scores were 50.0 [IQR 48.0; 51.0] points (preoperative assessment), 42.0 [IQR 38.0; 44.0] points (1st PACU assessment), 45.0 [IQR 42.5;47.0] points (2nd PACU assessment), and 45.0 [IQR 42.8;47.3] points (24 h assessment). Patients completed the questionnaires at a median time of 125.0 min [IQR 83.0; 156.8] after arrival in the PACU. The subgroup of patients, who underwent a second assessment in the PACU, completed the questionnaires at a median time of 189.0 min [IQR 148.8; 215.8]. Pre- and postoperative mean QoR-PACU scores for each item and the mean sum score are presented in Table 2. Figure 2 shows pre- and postoperative QoR-PACU scores.

Table 2 : Responsiveness

QoR-PACU item	Preoperative	Postoperative	Mean change [95% CI]	% change from baseline	Cohen effect size	Standardised reponse mean
**1**	3.7 ± 0.6	2.1 ± 1.2	-1.6 [-1.8; -1.5]	43.0	2.6	1.2
**2**	3.8 ± 0.5	2.3 ± 1.2	-1.6 [-1.7; -1.4]	39.0	3.2	1.2
**3**	3.7 ± 0.6	1.7 ± 1.1	-2.1 [-2.2; -1.9]	54.0	3.4	1.7
**4**	3.9 ± 0.4	3.6 ± 0.7	-0.3 [-0.4; -0.2]	7.7	0.7	0.4
**5**	4.0 ± 0.2	3.6 ± 0.8	-0.3 [-0.4; -0.3]	10.0	1.9	0.5
**6**	4.0 ± 0.1	4.0 ± 0.2	0.0 [-0.1; 0.0]	0.0	0.0	0.0
**7**	3.9 ± 0.4	3.7 ± 0.7	-0.2 [-0.3; -0.1]	5.1	0.5	0.3
**8**	4.0 ± 0.2	2.4 ± 1.2	-1.6 [-1.7; -1.4]	40.0	9.6	1.4
**9**	4.0 ± 0.2	3.5 ± 0.9	-0.5 [-0.6; -0.4]	12.0	2.2	0.6
**10**	3.4 ± 0.9	3.7 ± 0.6	0.3 [0.2; 0.5]	-8.8	-0.4	-0.3
**11**	3.7 ± 0.5	3.7 ± 0.6	-0.1 [-0.1; 0.0]	0.0	0.0	0.0
**12**	3.4 ± 0.6	3.2 ± 0.8	-0.2 [-0.3; -0.1]	5.9	0.3	0.2
**13**	3.8 ± 0.4	3.9 ± 0.4	0.0 [0.0; 0.1]	-2.6	-0.2	-0.2
**Sum**	49.0 ± 2.6	41.0 ± 5.0	-8.0 [-8.6; -7.4]	16.0	3.1	1.7

Table 2: Mean QoR-PACU scores for each item and the mean QoR-PACU sum score. Responsiveness is expressed with Cohen effect size (difference between preoperative and postoperative QoR-PACU scores, divided by the preoperative SD) and the standardized response mean (score difference divided by the SD of the score difference). Numbers are given as mean ± standard deviation (SD) unless otherwise indicated. QoR: Quality of recovery, PACU: postanesthesia care unit; CI: confidence interval.

Fig. 2: Responsiveness

Fig. 2. The radar chart – spider diagram shows mean scores of single items of the QoR-PACU preoperatively (green), in the PACU (red), at re-assessment in the PACU (purple), and on the day after surgery (pink). Each item of the questionnaire is presented as a spoke. The 5-point numeric rating scale is presented on the axis with numbers from 0 to 4.

Validity, reliability, and responsiveness

The comparison of postoperative QoR-PACU sum scores across categories of clinically revelant variables is presented in Table 3. The correlation between postoperative QoR-PACU sum scores and clinically relevant continous variables is presented in Figure 3. Cronbach's alpha was 0.67 (95% CI: 0.61 to 0.73), reflecting moderate internal consistency (32). The average of all split-half correlations was 0.52. The average of all split-half reliabilities was 0.69 ± 0.08. Interitem correlations and correlations between the QoR-PACU sum score and each item are presented in Figure 4 andS3 Table. There was a positive correlation between QoR-PACU score and the score at the second assessment approximately one hour later (r = 0.71, 95%CI: 0.37 to 0.88, p < 0.01) reflecting acceptable test-retest reliability. Cohen's effect size and standardized response mean are presented in Table 2.

Table 3 : Construct validity

**Variables**	**Postoperative QoR-PACU score**	***P***
**ASA physical status**		0.867
II (n=214)	42 [38;45]	
III (n=32)	43 [40;44]	
**OSAS[a]**		0.134
Low or intermediate risk (n=133)	41 [38;44]	
High risk or confirmed disease (n=91)	42 [40;45]	
**Premedication with midazolam**		0.247
no (n=238)	42 [38;44]	

yes (n=8)	44 [43;45]	
**Mode of intubation**		0.257
Direct langyroscopy (n=225)	42 [38;44]	
Video-assisted laryngoscopy (n=3)	46 [42;48]	
Switch from direct to video-assisted laryngoscopy (n=18)	42 [37;42]	
**Sevoflurane**		0.539
no (n=5)	39 [38;42]	
yes (n=241)	42 [38;44]	
**Propofol**		0.539
no (n=241)	42 [38;44]	
yes (n=5)	39 [38;42]	
**Depth of anaesthesia monitoring**		0.650
no (n=3)	42 [42;43]	
yes (n=243)	42 [38;45]	
**Antiemetic prophylaxis**		0.761
none (n=2)	39 [38;41]	
dexamethasone (n=6)	40 [37;44]	
ondansetron (n=3)	43 [38;46]	
dexamethasone + ondansetron (n=235)	42 [39;45]	

Table 3: Construct validity of categorical variables. Data are presented as median [25th; 75th percentile]. ASA: American Society of Anesthesiologists. OSAS: obstructive sleep apnea syndrome.

Fig 3: Construct validity

Fig 3. Correlation between postoperative QoR-PACU sum scores and clinically relevant continuous variables.

Fig 4: Inter-item correlation

Fig. 4. Correlation between each item and between the sum score and each item of the postoperative QoR-PACU using Spearman correlation coefficient.

Discussion

The aim of this study was to establish a questionnaire to assess self-reported QoR during the recovery period after elective non-cardiac surgery. We developed the QoR-PACU based on the 40-item QoR-40 questionnaire. We found high acceptability and feasibility with excellent recruitment and successful completion rates, good validity, adequate responsiveness, and moderate reliability.

A standardized tool for the assessment of patient reported QoR in the PACU is urgently needed for both research and clinical puroses. Myles et al emphasize that results of clinical research can only be considered valid if a reconfirmation is possible (8). However, comparability and impact of clinical research is substantially diminished by different outcome definitions and the use of numerous instruments for psychometric assessment (8,9,33). Therefore, it is important to standardize the endpoints in clinical research.
For decades the Aldrete scoring system has been widely used to determine, if a patient can be safely discharged from the PACU (20). Items addressed by the Aldrete score are limited to physical aspects: activity, respiration, circulation, consciousness and color / oxygen saturation (21,34). However, the definition of adequate recovery may differ substantially between patients and caregivers. Including the patients' perception of postoperative recovery immediately after surgery provides a basis for the optimization of recovery, which may result in better outcomes and might even have a beneficial effect on length of stay and healthcare costs (3).

We chose clinically relevant aspects of intra- and postoperative care to assess construct validity. Pain intensity, piritramide dose, and PACU length of stay negatively correlated with QoR in the PACU. Our results confirm that pain perception plays a substantial role in perioperative care with a major impact on patient's perception of health status and recovery (35). Despite homogeneity of surgical procedures we found a large range of PACU length of stay. Shorter stay in the PACU

correlated with better QoR, which confirms previous reports on the association between PACU length of stay and postoperative complications (36).

Psychometric properties of the QoR-PACU reveiled good validity and adequate responsiveness however, measures of reliability including internal consistency were moderate. The fact that the internal consistency of the QoR-PACU was not as high as expected is interesting, since all items of the QoR-PACU were derived from the QoR-40 which has been developed to evaluate the quality of recovery 24 hours after surgery and has been validated extensively. Items showing high validity and reliability 24 hours after surgery showed only sufficient internal consistency in the immediate postoperative period. Several factors may account for the difference in internal consistency. First and most importantly, the patients' mental and physical condition changes rapidly during the early postoperative period. Of note, vigilance and pain perception are interconnected. When the effects of anesthesia and analgesia wear off, consciousness improves and patients become more susceptible to postoperative pain. Therefore, it is resonable to expect that self-perceived recovery in the early postoperative period will change substantially within a 30-minute time frame. Second, we noticed that the simultaneous application of measures of frequency, as used in the QoR-PACU, and measures of intensity, as used in the NRS, during the recovery period led to confusion with study participants. We tried to avoid this problem by reducing the 11-point scale to a 5-point scale linked with adverbs of frequency. Yet, difficulty in understanding measures of frequency might have influenced our results. Third, it is noteworthy that we observed a relatively small change from preoperative to PACU scores in our study population. The mean change from preoperative to PACU scores was <0.5 for 9 items, but not for the features pain, feeling confused, dry mouth, and sore throat. The majority of patients had a rather low perioperative risk as reflected by the ASA physical status. High perioperative risk has been found to be associated with poor recovery after colorectal cancer surgery (37). Similary, low ASA physical status might have contributed to the overall high QoR reported by participants of our study.

We found the application of the QoR-PACU during the recovery period feasible with high response and completion rates. Sum scores were highest at baseline on the day before surgery and lowest during assessment in the PACU followed by an increase on the first postoperative day. The development of QoR-PACU sum scores over time indicates that the QoR-PACU adequately mirrors QoR despite moderate internal consistency.

This validation study was performed at the PACU of a prostate cancer clinic. All surgical procedures and perioperative care at our prostate cancer center are highly standardized. Although allowing for excellent comparability between participants, generalizability is limited. We included solely male patients scheduled for radical prostatectomy. Results from previous studies suggest that gender aspects have an impact on postoperative QoR and speed of recovery. Overall, female patients tend to have lower QoR and longer PACU stay (2,38,39). Morevover, pain intensity, nausea, and vomiting after surgery are more frequently reported by female patients (38,40). Gender aspects may be of high importance in individualized perioperative care and postoperative recovery.

Future studies should evaluate the psychometric properties of the QoR-PACU in a more heterogenous patient population, including female and gender diverse patients, as well as a greater variety of patient-related and procedure-related risk factors. This might reveal, whether the issue of moderate internal consistency was primarily linked to the characteristics of the initial study cohort, or whether items have to be revised substantially to be suitable for patients in the PACU. For the modification of the QoR-PACU, it might be helpful to consider suggestions from patients and caregivers.

Conclusion

This study presents the development of a questionnaire to assess self-reported QoR after surgery in the PACU. We found excellent feasibility, good validity and adequate responsiveness. Against our hypothesis, we did not find a high internal consistency of the QoR-PACU. In future studies, the QoR-PACU should be evaluated in more heterogeneous patient populations including female and gender-diverse patients with varying degress of perioperative risk.

Acknowledgements

The authors thank Lars Nawrath for his support in the process of selecting items of the QoR-40 questionnaire to develop the first version of the QoR-PACU.

All authors approved of the version to be published and agree to be accountable for all aspects of the work, thereby ensuring that questions related to the accuracy or integrity of any part of the work are appropriately investigated and resolved.

References

1. Myles PS, Hunt JO, Nightingale CE, Fletcher H, Beh T, Tanil D, et al. Development and psychometric testing of a quality of recovery score after general anesthesia and surgery in adults. Anesth Analg. 1999 Jan;88(1):83–90.

2. Myles PS, Weitkamp B, Jones K, Melick J, Hensen S. Validity and reliability of a postoperative quality of recovery score: the QoR-40. Br J Anaesth. 2000 Jan;84(1):11–5.

3. Borrell-Vega J, Humeidan ML, Bergese SD. Defining quality of recovery - What is important to patients? Best Pract Res Clin Anaesthesiol. 2018 Dec;32(3–4):259–68.

4. Myles PS. More than just morbidity and mortality - quality of recovery and long-term functional recovery after surgery. Anaesthesia. 2020 Jan;75 Suppl 1:e143–50.

5. Shulman M, Myles P. Measuring perioperative outcome. Curr Opin Anaesthesiol. 2016 Dec;29(6):733–8.

6. Neville A, Lee L, Antonescu I, Mayo NE, Vassiliou MC, Fried GM, et al. Systematic review of outcomes used to evaluate enhanced recovery after surgery. Br J Surg. 2014 Feb;101(3):159–70.

7. Stark PA, Myles PS, Burke JA. Development and psychometric evaluation of a postoperative quality of recovery score: the QoR-15. Anesthesiology. 2013 Jun;118(6):1332–40.

8. Myles PS, Grocott MPW, Boney O, Moonesinghe SR, COMPAC-StEP Group. Standardizing end points in perioperative trials: towards a core and extended outcome set. Br J Anaesth. 2016 May;116(5):586–9.

9. Myles PS, Boney O, Botti M, Cyna AM, Gan TJ, Jensen MP, et al. Systematic review and consensus definitions for the Standardised Endpoints in Perioperative Medicine (StEP) initiative: patient comfort. Br J Anaesth. 2018 Apr;120(4):705–11.

10. Abola RE, Bennett-Guerrero E, Kent ML, Feldman LS, Fiore JF, Shaw AD, et al. American Society for Enhanced Recovery and Perioperative Quality Initiative Joint Consensus Statement on Patient-Reported Outcomes in an Enhanced Recovery Pathway. Anesth Analg. 2018 Jun;126(6):1874–82.

11. Vignaud M, Morel C, Henault A, Futier E, Pereira B, Lambert C, et al. Variability and reliability of the French version of the Quality of Recovery-40 Questionnaire (QoR-40). Anaesth Crit Care Pain Med. 2021 Apr;40(2):100822.

12. Chen Y, Wang J, Liu S, Lai W, Liu J, Wang Z, et al. Development and Validation of the Chinese Version of the Quality of Recovery-40 Questionnaire. Ther Clin Risk Manag. 2020;16:1165–73.

13. Lee JH, Kim D, Seo D, Son JS, Kim DC. Validity and reliability of the Korean version of the Quality of Recovery-40 questionnaire. Korean J Anesthesiol. 2018 Dec;71(6):467–75.

14. Terkawi AS, Myles PS, Riad W, Nassar SN, Mahmoud M, AlKahtani GJ, et al. Development and validation of Arabic version of the postoperative quality of recovery-40 questionnaire. Saudi J Anaesth. 2017 May;11(Suppl 1):S40–52.

15. Kleif J, Edwards HM, Sort R, Vilandt J, Gögenur I. Translation and validation of the Danish version of the postoperative quality of recovery score QoR-15. Acta Anaesthesiol Scand. 2015 Aug;59(7):912–20.

16. Demumieux F, Ludes PO, Diemunsch P, Bennett-Guerrero E, Lujic M, Lefebvre F, et al. Validation of the translated Quality of Recovery-15 questionnaire in a French-speaking population. Br J Anaesth. 2020 Jun;124(6):761–7.

17. Kim D, Kim JK, Yeo J. Translation and Validation of the Korean Version of the Postoperative Quality of Recovery Score QoR-15. BioMed Res Int. 2020;2020:3456234.

18. Kahl U, Gebhardt N, Brodersen K, Kainz E, Schirren L, Yu Y, et al. Validation of a translated Quality of Recovery-15 questionnaire in German patients undergoing elective noncardiac surgery. Br J Anaesth. 2021 Nov;127(5):e161–3.

19. Picconi E, Iacobucci T, Adducci E, Gualtieri E, Beccia G, Sollazzi L. Translation and validation of the Italian version of the postoperative quality of recovery score QoR-15. Minerva Anestesiol. 2020 Jul;86(7):787–9.

20. Nakatani S, Ida M, Tanaka Y, Okamoto N, Wang X, Nakatani H, et al. Translation and validation of the Japanese Version of the Quality of Recovery-15 Questionnaire. J Anesth. 2021 Jun;35(3):426–33.

21. Aldrete JA, Kroulik D. A postanesthetic recovery score. Anesth Analg. 1970 Dec;49(6):924–34.

22. Sá AC, Sousa G, Santos A, Santos C, Abelha FJ. Quality of Recovery after Anesthesia: Validation of the Portuguese Version of the ‘Quality of Recovery 15’ Questionnaire. Acta Med Port. 2015 Oct;28(5):567–74.

23. Miklić Bublić M, Miklić P, Barl P, Matas M, Sekulić A. CROATIAN VERSION OF THE QUALITY OF RECOVERY QUESTIONNAIRE (QoR-40): TRANSCULTURAL ADAPTATION AND VALIDATION. Acta Clin Croat. 2021 Jun;60(2):237–45.

24. Yaghoobi S, Hamidfar M, Lawson DM, Fridlund B, Myles PS, Pakpour AH. Validity and reliability of the Iranian version of the quality of recovery-40 questionnaire. Anesthesiol Pain Med. 2015 Apr;5(2):e20350.

25. Ludbrook G, Lloyd C, Story D, Maddern G, Riedel B, Richardson I, et al. The effect of advanced recovery room care on postoperative outcomes in moderate-risk surgical patients: a multicentre feasibility study. Anaesthesia. 2021 Apr;76(4):480–8.

26. Boateng GO, Neilands TB, Frongillo EA, Melgar-Quiñonez HR, Young SL. Best Practices for Developing and Validating Scales for Health, Social, and Behavioral Research: A Primer. Front Public Health. 2018;6:149.

27. Nunnally J. Pyschometric Theory. 1978.

28. Clark LA, Watson D. Constructing validity: Basic issues in objective scale development. Psychol Assess. 1995 Sep;7(3):309–19.

29. Vetter TR, Cubbin C. Psychometrics: Trust, but Verify. Anesth Analg. 2019 Jan;128(1):176–81.

30. Revelle W. psych: Procedures for Psychological, Psychometric, and Personality Research [Internet]. 2021 [cited 2022 May 1]. Available from: https://CRAN.R-project.org/package=psych

31. Sherman RA. multicon: Multivariate Constructs [Internet]. 2015 [cited 2022 May 1]. Available from: https://CRAN.R-project.org/package=multicon

32. Taherdoost H. Validity and Reliability of the Research Instrument; How to Test the Validation of a Questionnaire/Survey in a Research. SSRN Electron J [Internet]. 2016 [cited 2022 Jun 20]; Available from: https://www.ssrn.com/abstract=3205040

33. Bowyer A, Royse C. The importance of postoperative quality of recovery: influences, assessment, and clinical and prognostic implications. Can J Anaesth J Can Anesth. 2016 Feb;63(2):176–83.

34. Aldrete JA. The post-anesthesia recovery score revisited. J Clin Anesth. 1995 Feb;7(1):89–91.

35. Nimmo SM, Foo ITH, Paterson HM. Enhanced recovery after surgery: Pain management. J Surg Oncol. 2017 Oct;116(5):583–91.

36. Mann-Farrar J, Egan E, Higgins A, Wysocki L, Vaux A, Arndell E, et al. Are Postoperative Clinical Outcomes Influenced by Length of Stay in the Postanesthesia Care Unit? J Perianesthesia Nurs Off J Am Soc PeriAnesthesia Nurses. 2019 Apr;34(2):386–93.

37. Jakobsson J, Idvall E, Kumlien C. Patient characteristics and surgery-related factors associated with patient-reported recovery at 1 and 6 months after colorectal cancer surgery. Eur J Cancer Care (Engl). 2017 Nov;26(6).

38. Buchanan FF, Myles PS, Cicuttini F. Effect of patient sex on general anaesthesia and recovery. Br J Anaesth. 2011 Jun;106(6):832–9.

39. Myles PS, McLeod AD, Hunt JO, Fletcher H. Sex differences in speed of emergence and quality of recovery after anaesthesia: cohort study. BMJ. 2001 Mar 24;322(7288):710–1.

40. Buchanan FF, Myles PS, Cicuttini F. Patient sex and its influence on general anaesthesia. Anaesth Intensive Care. 2009 Mar;37(2):207–18.

Supporting information

S1 Table. QoR-PACU Version 3.0 preoperative.

S2 Table. QoR-PACU Version 3.0 postoperative.

S3 Table. Postoperative interitem correlation.

S3 Table: Postoperative assessment: Interitem correlations for the 13 items of the QoR-PACU score. Correlations are expressed as Pearson correlation coefficients.

Figure 1

Click here to access/download;Figure;PACQOR 3 Figure 1 flowchart 2022-05-01.pptx

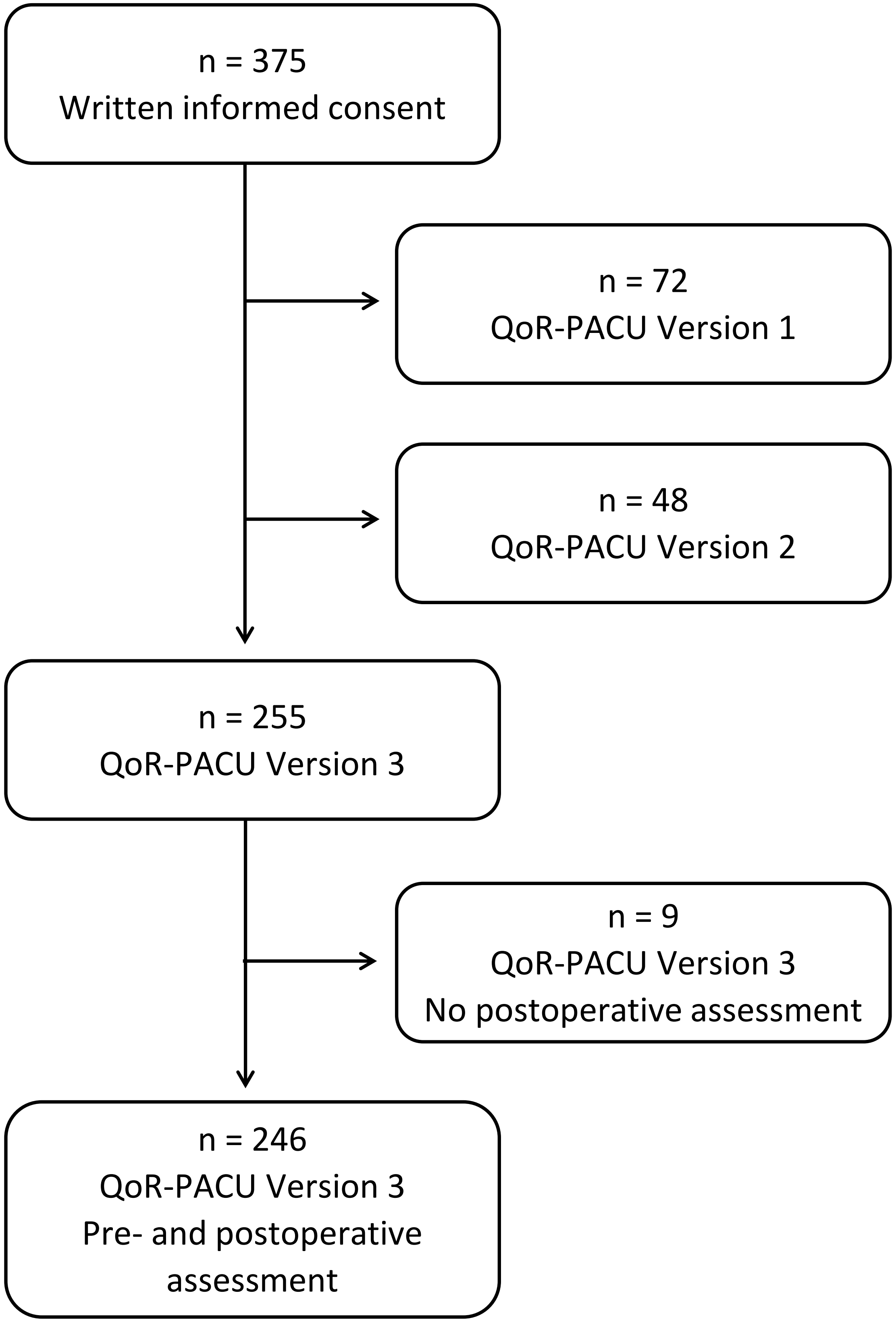

Figure 2

Click here to access/download;Figure;PACQOR 3 Figure 2 spider 2021-08-26.pdf

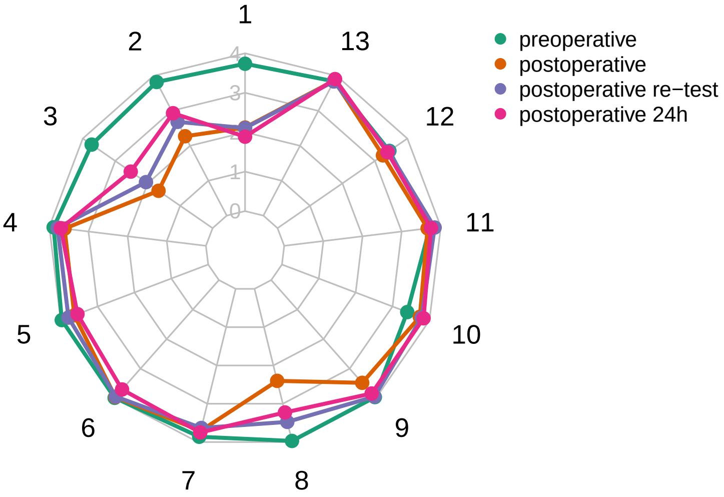

Figure 3

Click here to access/download;Figure;PACQOR 3

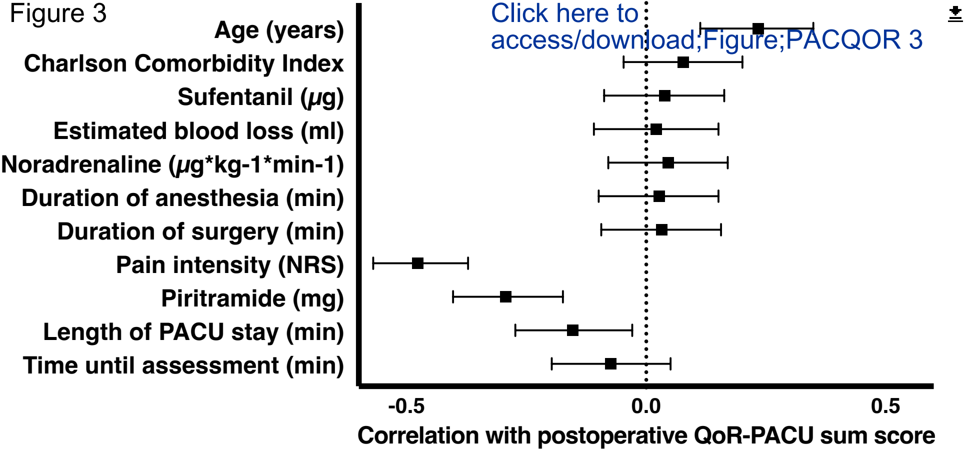

Figure 4

Click here to access/download;Figure;PACQ

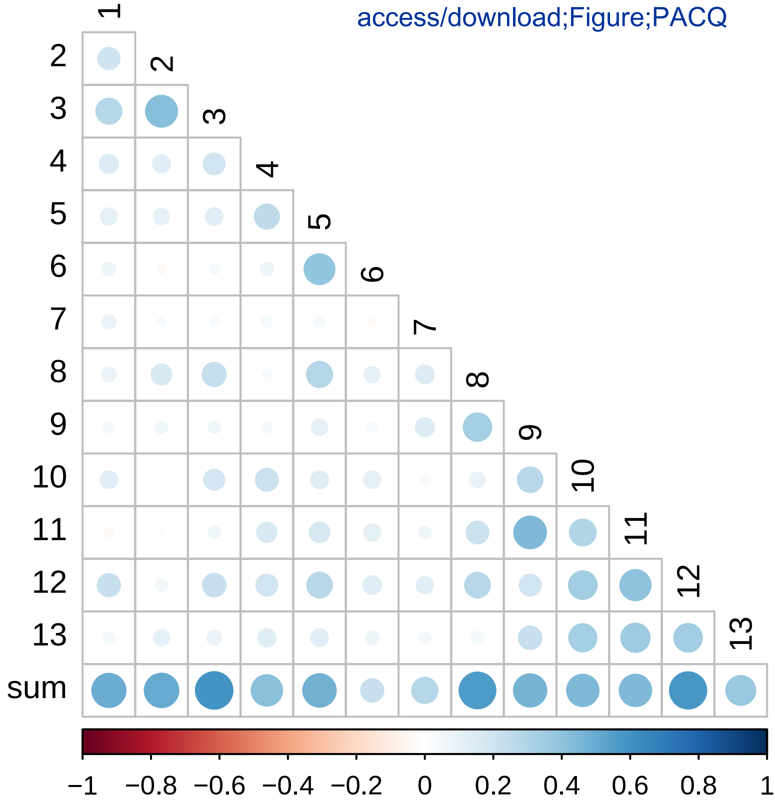

Supplementary Table 1

Click here to access/download
Supporting Information
Supplementary_Table_1_070722.docx

Supplementary Table 2

Click here to access/download
Supporting Information
Supplementary_Table_2_070722.docx

Supporting Information S3 Table

Click here to access/download
Supporting Information
S3_Table.docx
