# Supplementary material for: Psychometric evaluation of a quality of recovery score for the postanesthesia care unit—A preliminary validation study
Source: PLoS One. 2023 Aug 15;18(8):e0289685. doi: 10.1371/journal.pone.0289685 (PMC10426991; doi:10.1371/journal.pone.0289685)
Supplement: Attachment — Submitted filename: R1_Point-by-point_response_180922.docx [file pone.0289685.s005.docx]

Psychometric evaluation of a quality of recovery score for the postanesthesia care unit – a prospective validation study.

**Point by Point Response to the reviewers’ comments.**

*We thank the reviewers for taking the time to read and process our manuscript. We have integrated the reviewers’ suggestions and believe that this has substantially improved the manuscript.*

*Please find below the reviewers’ comments written upright in black and our responses written in blue italics.*

**Reviewer #1:**

1. There is smaller sample size than calculated. It should be clarified.

*We thank the reviewer for alerting us to this. According to the suggestions of both reviewer 1 and 2, we have amended the methods section for the following explanation:*

*“There is no consistent recommendation regarding sample size for the development and the evaluation of a questionnaire. A “rule of thumb” suggests at least 10 participants for each scale item (Boateng et al. 2018; Nunnally 1978). This would result in 160 participants for the 16-items version 1, or 130 participants for the 13-items version 3 of the questionnaire. We did not a priori decide on the exact number of items to be included in the questionnaire. Instead, we planned to conduct a pilot phase to optimize the development of the questionnaire. Therefore, we opted for the inclusion of 375 patients, allowing for an extensive pilot testing sample as well as a large drop-out rate.*

*See revised article with highlighted changes, p.7*

2. One more area is missing, the reasons that compelled the patients to fail to reach the follow up visits are to be evaulatied.

*We thank the reviewer for alerting us to this. Due to postponed surgeries, 9 patients could not be met in the PACU by the study team. We have included this information in the flow chart (Fig 1).*

*See revised Fig 1*

**Reviewer #2:**

1. my main concern is how a tool in German language could be of interest to wider international audience where English is the primary language.

*We agree with the reviewer that in order to be useful to an international public, the QoR-PACU needs to be translated to English and subsequently to various more languages. It is a natural limitation that any questionnaire can only be used with patients who have excellent understanding of the respective language* *the questionnaire was written in. We developed the QoR-PACU in German language because we wanted to validate and use the instrument in our German speaking patient population. We have added these thoughts to the discussion section of the manuscript.*

*See revised article with highlighted changes, p.19*

2. Another main concern is testing in PACU –120 minutes after PACU admission seems late as many PACU stays are much shorter for this surgical procedure.

*We agree with the reviewer that this topic merits further discussion.*

*In our study, the overall length of stay in our patient cohort was rather long with a median time of 152 minutes. Data on PACU length of stay varies widely across studies:*

- *A retrospective analysis of 17.047 patients revealed a median PACU length of stay of 117 (IQR 82, 165) minutes (Weissman, Scemama, and Weiss 2019).*
- *A study investigating the relationship of pain and postoperative nausea and vomiting with the PACU length of stay in 9.603 patients, showed a mean PACU length of stay of 3.2 ± 2.3 hours (Ganter et al. 2014).*
- *A study investigating* *whether BIS-guided anaesthesia reduced the incidence of awareness during surgery in adults, found a median PACU length of stay of 63 (40–95) min in 1.225 patients with BIS monitoring and 66 (40–100) min in 1.238 patients without BIS monitoring (Myles et al. 2004).*

*In order for the instrument to be suitable for patients with shorter PACU stays, it is necessary to re-evaluate the QoR-PACU earlier after arrival in the PACU.*

*We have included the following paragraph in the discussion:*

*“We assessed patients 120 ± 60 minutes after arrival in the PACU. This long period of recovery may have enhanced the understanding and completion of the questionnaire. However, in many anesthetic and surgical departments, it is common for patients to leave the PACU earlier (Myles et al. 2004). Future studies should evaluate whether the QoR-PACU is also feasible earlier after arrival in the PACU.”*

*See revised article with highlighted changes, p.17*

Abstract:
Needs to be improved based on the comments below. Additionally add:

- refer to German tool,

*We added this information to the abstract.*

*See revised article with highlighted changes, p.2*

- sample size is 255, others were in piloting stage of the tool development;

*We added the desired information: “We included 375 patients. After two piloting phases including 72 and 48 patients, respectively, we administered the final version of the QoR-PACU to 255 patients, with a completion rate of 96.5%.”*

*See revised article with highlighted changes, p.2*

3. Introduction:
   Overall, well described background. I suggest you improve the last sentence: "in patients after general anesthesia for elective non-cardiac surgery." – this is misleading as your tool was used only for patients undergoing urologic surgery and in German language only.

*We have included the following sentence:” […] Therefore we developed the German QoR-PACU and evaluated its psychometric properties in a cohort of patients scheduled for elective urologic surgery.”*

*See revised article with highlighted changes, p.4*

4. Material and methods:

I wonder if quaternary (check spelling for this word please) hospital care is readily generalizable (see definitions of tertiary – quaternary care); your study was registered and there you mentioned tertiary care hospital (also the radical prostatectomy is a common procedure)

*We thank the reviewer for alerting us to this. Our institution includes departments which provide quaternary care. However, we agree that the procedure of prostatectomy (although performed at a highly specialized prostate cancer center) is better referred to as tertiary care. We have changed this term accordingly.*

*See revised article with highlighted changes, p.5*

5. This sentence is unclear, including chronologically

– is this during the instrument development stage? How were the patients randomly selected? Or was this an opportunistic sample? Maybe this should be put in the next section? "We performed a pre-test of the QoR-PACU in a randomly selected cohort of 10 patients to assess feasibility."

*We agree with the reviewer that this sentence belongs to the development section. We have changed this in the manuscript. Additionally, we changed the term “randomly selected cohort” to “convenience sample”.*

*See revised article with highlighted changes, p.6*

6. Please take care of writing this: 60 minutes ± 30: should be 60 ± 30 minutes

*We have changed this in the manuscript.*

*See revised article with highlighted changes, p.5*

7. In the section: Development and adaptation of the QoR-PACU; I suggest you refer to pilot 1 and pilot 2 instead of early study period: "After a successful pretest phase, we administered the 16-item QoR-PACU (version 1) to 72 patients – the first pilot testing of the QoR-PACU." And similar where needed in this paragraph

*We thank the reviewer for this suggestion. We have rephrased this in the manuscript.*

*See revised article with highlighted changes, p.6*

8. Sample Size: I suggest you use only 10 participants per item here and add that you aimed for at least 160 participants with full completion of the questionnaire version 3 (you did not reach 375 that you mention here).

*We are grateful for this pragmatic suggestion. We have changed the paragraph as follows to the following explanation:*

*“There is no consistent recommendation regarding sample size for the development and the evaluation of a questionnaire. A “rule of thumb” suggests at least 10 participants for each scale item (Boateng et al. 2018; Nunnally 1978). This would result in 160 participants for the 16-items version 1, or 130 participants for the 13-items version 3 of the questionnaire. We did not a priori decide on the exact number of items to be included in the questionnaire. Instead, we planned to conduct a pilot phase to optimize the development of the questionnaire. Therefore, we opted for the inclusion of 375 patients, allowing for an extensive pilot testing sample as well as a large drop-out rate”*

*See revised article with highlighted changes, p.7*

9. What I am confused about is several subdivision into subgroups for different testing... you should define sample sizes for all of these here or at least further develop figure 1 in results to show the numbers of patients doing reassessments

*Following the suggestion of the reviewer, we now present these two subsets of patients in figure 1.*

*See Fig1 and table 2*

10. I wonder if you chose the items from the QoR40 tool from English or valid German translation? Please clarify in the methods.

*We have selected the 15 items from the validated German translation of the QoR-40 (Plöger 2008). We have now specified this in the Development section.*

*See revised article with highlighted changes, p.6*

11. Responsiveness definition: ability to detect clinical important change: what does this mean for your data?
12. Add in table 2 which is important change?

*We thank the reviewer for alerting us to this critical issue. Indeed, the term “clinically important change” is problematic, as this would require the comparison with a gold standard (Middel et al., n.d.; Terwee et al. 2007). Regarding the QoR-PACU this is not possible, since* *there hasn’t been any tool for the measurement of patient-reported recovery in the PACU to date.* *The correlation of QoR-PACU sum scores with clinically important parameters, such as patient reported pain intensity (r= -0.48, 95%CI: -0.57 to -0.37, p <0.001) confirms the clinical relevance of the observed change in QoR-PACU sum scores. Since we did not analyse the correlation of single items with clinically important parameters, we rephrased the responsiveness definition as follows: “Responsiveness refers to the ability of the instrument to detect change over time.”*

*See revised article with highlighted changes, p.8*

*Additionally, we have added the following paragraph as a limitation:*

*“**When evaluating responsiveness, it is commendable to compare the ability of the instrument to detect change to a gold standard (32,41). Unfortunately, in the present study this was not possible, since there hasn’t been any tool for the measurement of patient-reported recovery in the PACU to date. We followed the approach of Stark et al. and evaluated the ability of the QoR-PACU to detect change over time based on the Cohen’s effect size and the standardized response mean (7). Additionally, the correlation of QoR-PACU sum scores with clinically important parameters, such as patient-reported pain intensity confirms the clinical relevance of the observed change in QoR-PACU sum scores.”*

*See revised article with highlighted changes, p.18*

*To enable the appraisal of Cohen’s effect size, we’ve added the Cohen thresholds small (0.2), medium (0.5) and large (0.8) (Norman, Wyrwich, and Patrick 2007) to the legend of table 3.*

*See revised table 3*

13. This is unclear: "The proportion of patients who successfully completed the QoR-PACU postoperatively was used to assess acceptability and feasibility." What proportion? How was this tested? Definitions?

*We have clarified this terminology throughout the manuscript.*

*We have assessed acceptability of the instrument based on interviews with patients and PACU staff during the piloting and the testing phase. We did not quantify results of these interviews; however, we implemented their feedback in the development of version 3 of the QoR PACU.*

*We used the percentage of successfully completed pre- and postoperative QoR-PACU questionnaires to assess feasibility. The completion rate was 96.5%. We therefore concluded, the assessment of patient reported recovery in the PACU with the QoR PACU is highly feasible*

*See revised article with highlighted changes, p.6, p.9*

14. Results:

Please see above abour improving figure 1

*We have revised figure 1.*

*See revised Fig 1*

15. Table 2:

- i am ok with means here, however you should improve statistical part of the methods to reflect that as you say only medians with IQR will be reported;

*We have added this information in the statistical part of the methods.*

*See revised article with highlighted changes, p.8*

- %Change from baseline: be careful which should be negative – almost all?

*While the mean change is displayed as a negative or positive value, the % change from baseline is generally not displayed with an algebraic minus- or plus-sign. Please see for comparison Stark et al.* (Stark, Myles, and Burke 2013)*.*

- Also the paragraph before this table reports medians and then the table reports means – should be consistent;

*We decided to report mainly medians, because we believe that it is the most appropriate way to report questionnaire results. However, the calculation of the standardised response mean is based on mean values, hence the inconsistency between the text and the table.*

*We have added the median sum scores of the four QoR-PACU assessments with the respective number of patients and time of assessment as Table 2.*

*See revised table 2*

- Also: the table title Responsiveness is not ok; please improve to XYZ and responsiveness.
  *Following the approach of Stark et al.* (Stark, Myles, and Burke 2013)*, we changed the title to “Change in mean QoR-PACU scores from preoperative to postoperative PACU assessment”.*

*See revised table 3*

16. Figure 2: also add n of patients in brackets in the legend of the four colors; also improve figure 2 title

*We included this information in the figure caption and changed the title to “Change in mean QoR-PACU scores”.*

*See revised Fig 2*

17. Table 3: I suggest you remove this table completely as the construct validity in this context is not valid: your numbers of patients in each of the category are far from comparable in all clinically relevant variable as you say with the exception of N for ASA and OSAS (but I also disagree with the notion how your tool would be connected to mode of intubation for example). Also modify methods section appropriately

*We thank the reviewer for this suggestion. We have deleted this table. We now present information on the two variables ASA and OSAS in the main text, since we do not want to omit negative results. Additionally, this information might be interesting for comparison with future studies.*

*See revised article with highlighted changes, p.13*

18. Figure 3: I am fine with this one; please add what is presented in description: mean with 95% CI?? What statistical test was used for correlations – please also add this in the figure

*We have included this information in the figure caption.*

*See revised Fig 3*

19. Discussion:
    Some parts need to be modified to reflect my comments above including the first paragraph which needs to be significantly improved.

- How do you conclude this Is highly feasible and acceptable?

*Please see above (Reviewer 2, Point 13)*

*See revised article with highlighted changes, p.6, p.9*

- Add the earliest 2 hours after PACU admission?

*We added this information.*

*See revised article with highlighted changes, p.16*

- Omit excellent recruitment this is ambiguous; leave high completion rates;

*We changed the sentence as follows: “We found high acceptability and feasibility, good validity, adequate responsiveness, and moderate reliability.”*

*See revised article with highlighted changes, p.16*

- also clarify in the methods section if patients alone completed the surveys or were the items read to them and explained?

*We clarified this section as follows: “Patients read and completed the questionnaire themselves. If necessary, patients were provided with glasses by the study team.”*

*See revised article with highlighted changes, p.5*

- Your PACU times are interesting as in many countries patients mostly leave PACUs after around 60 minutes – please add to limitations how can your QoR instrument be used sooner;

*Please see above (Reviewer 2, Point 2).*

*See revised article with highlighted changes, p.17*

- Use some citations when you refer to gender influences in the discussion

We have added the appropriate citations:

*2. Myles PS, Weitkamp B, Jones K, Melick J, Hensen S. Validity and reliability of a postoperative quality of recovery score: the QoR-40. Br J Anaesth. 2000 Jan;84(1):11–5.*

*39. Buchanan FF, Myles PS, Cicuttini F. Effect of patient sex on general anaesthesia and recovery. Br J Anaesth. 2011 Jun;106(6):832–9.*

*40. Buchanan FF, Myles PS, Cicuttini F. Patient sex and its influence on general anaesthesia. Anaesth Intensive Care. 2009 Mar;37(2):207–18.*

*41. Myles PS, McLeod AD, Hunt JO, Fletcher H. Sex differences in speed of emergence and quality of recovery after anaesthesia: cohort study. BMJ. 2001 Mar 24;322(7288):710–1.*

*See revised article with highlighted changes, p.18-19*

***References***

*Boateng, Godfred O., Torsten B. Neilands, Edward A. Frongillo, Hugo R. Melgar-Quiñonez, and Sera L. Young. 2018. ‘Best Practices for Developing and Validating Scales for Health, Social, and Behavioral Research: A Primer’. Frontiers in Public Health 6 (June): 149. https://doi.org/10.3389/fpubh.2018.00149.*

*Ganter, Michael T, Stephan Blumenthal, Seraina Dübendorfer, Simone Brunnschweiler, Tim Hofer, Richard Klaghofer, Andreas Zollinger, and Christoph K Hofer. 2014. ‘The Length of Stay in the Post-Anaesthesia Care Unit Correlates with Pain Intensity, Nausea and Vomiting on Arrival’. Perioperative Medicine 3 (November): 10. https://doi.org/10.1186/s13741-014-0010-8.*

*Middel, Berrie, Hanna Kuipers-Upmeijer, Jelte Bouma, Michiel Staal, Dettie Oenema, Theo Postma, Sijmon Terpstra, and Roy Stewart. n.d. ‘EVect of Intrathecal Baclofen Delivered by an Implanted Programmable Pump on Health Related Quality of Life in Patients with Severe Spasticity’, 6.*

*Myles, PS, K Leslie, J McNeil, A Forbes, and MTV Chan. 2004. ‘Bispectral Index Monitoring to Prevent Awareness during Anaesthesia: The B-Aware Randomised Controlled Trial’. The Lancet 363 (9423): 1757–63. https://doi.org/10.1016/S0140-6736(04)16300-9.*

*Norman, G. R., Kathleen W. Wyrwich, and Donald L. Patrick. 2007. ‘The Mathematical Relationship among Different Forms of Responsiveness Coefficients’. Quality of Life Research 16 (5): 815–22. https://doi.org/10.1007/s11136-007-9180-x.*

*Nunnally, Jum C. 1978. Psychometric Theory. 2d ed. McGraw-Hill Series in Psychology. New York: McGraw-Hill.*

*Plöger, Birgit. 2008. ‘Validierung einer deutschen Übersetzung des Quality-of-Recovery-Scores-40 (QoR-40) als Maß der patientenzentrierten postoperativen Ergebnisqualität’. Marburg: Philipps-Universität Marburg.*

*Sivan, Manoj. 2009. ‘Interpreting Effect Size to Estimate Responsiveness of Outcome Measures’. Stroke 40 (12): e709–e709. https://doi.org/10.1161/STROKEAHA.109.566836.*

*Stark, Peter A., Paul S. Myles, and Justin A. Burke. 2013. ‘Development and Psychometric Evaluation of a Postoperative Quality of Recovery Score’. Anesthesiology 118 (6): 1332–40. https://doi.org/10.1097/ALN.0b013e318289b84b.*

*Terwee, Caroline B., Sandra D. M. Bot, Michael R. de Boer, Daniëlle A. W. M. van der Windt, Dirk L. Knol, Joost Dekker, Lex M. Bouter, and Henrica C. W. de Vet. 2007. ‘Quality Criteria Were Proposed for Measurement Properties of Health Status Questionnaires’. Journal of Clinical Epidemiology 60 (1): 34–42. https://doi.org/10.1016/j.jclinepi.2006.03.012.*

*Weissman, Charles, Jeremy Scemama, and Yoram G. Weiss. 2019. ‘The Ratio of PACU Length-of-Stay to Surgical Duration: Practical Observations’. Acta Anaesthesiologica Scandinavica 63 (9): 1143–51. https://doi.org/10.1111/aas.13421.*
