# Supplementary material for: Psychometric evaluation of a quality of recovery score for the postanesthesia care unit—A preliminary validation study
Source: PLoS One. 2023 Aug 15;18(8):e0289685. doi: 10.1371/journal.pone.0289685 (PMC10426991; doi:10.1371/journal.pone.0289685)
Supplement: Attachment — Submitted filename: R2_response_to_reviewers_300623.docx [file pone.0289685.s006.docx]

**PONE-D-22-18272R1 – Revision 2**

Reviewer's Responses to Questions

Reviewer #3:

1. As one of the reviewers commented, it is true that the main limitation is that the instrument is in German and adapted to the context of the country, but since there are no instruments for assessing health outcomes in perioperative patients, I consider its publication to be relevant, allowing later cultural adaptation to other languages.

*With this study, we aimed to develop a new tool for the assessment of patient-reported quality of recovery (QoR) in the postanesthesia care unit (PACU). A questionnaire can only be used with patients who have excellent understanding of the respective language* *the questionnaire was written in. Therefore, to evaluate and to use the instrument in our German speaking patient population, we developed the QoR-PACU in German language.*

*We hope that international psychometricians and clinicians will find the QoR-PACU useful and will translate it into other languages.*

*We have added these considerations to the discussion section of the manuscript.*

*See revised article with highlighted changes, p.19*

2. As a general comment, they mention that there are multiple instruments for assessing recovery 24 hours after the intervention, but instead of reviewing these instruments or generating a pool of items, they decide to use the QoL-40, and it would be convenient to justify this aspect.

*We agree with the reviewer that using a larger pool of items to choose from, might have benefited the development of the QoR-PACU. Therefore, we have added this aspect as a limitation. Yet, we have explicitly decided to derive the QoR-PACU from the QoR questionnaires, since their use has been recommended by the StEP inititiative* [1]*. Future studies should include suggestions by anaesthesiologists, PACU nurses and patients.*

*Revised manuscript with highlighted changes, p.19*

3. Regarding the abstract, perhaps it would be good to include the time in which the patients answered the instrument before and after the intervention (which seems to be about 2h after the intervention).

*We have added this information to the abstract.*

*Revised manuscript with highlighted changes, p.2*

4. In the methods section, it would be good to indicate how the subsamples were selected to assess test-retest reliability, as well as for the pilot studies and why this sample size.

***Pilot study selection***

*The patients who were included in the pilot testing phase were selected consecutively in analogy to the patients included in the main analysis. All patients who met the inclusion criteria and did not meet exclusion criteria were approached by a member of the study team for participation in the study.*

***Pilot study sample size***

*We did not calculate sample sizes for the pilot study. Instead, the pilot studies were conducted in exactly the same way as the main study. The pilot phase was halted when we encountered problems that led us to modify the questionnaire.*

*The first pilot phase was halted after 72 patients, when we noticed misunderstandings regarding similarity between the 11-point response scale (measure of frequency) and the numeric rating scale used for assessment of pain (measure of intensity). The second pilot phase was halted after 48 patients, when we noticed that the font size of the questionnaire was too small for some patients.*

***Test-retest subgroup selection***

*To assess test-retest reliability, 19 patients completed the QoR-PACU twice, 60 ± 30 minutes after the first assessment. The two assessments were conducted by two different raters. On 5 consecutive days, two members of the study team were available for testing. During these days, all study participants underwent a second assessment and were included for test-retest reliability.*

***Test-Retest sample size***

*We aimed to analyze test-retest reliability in 20-25 patients* [2–4]*. As explained above, we performed retest on 5 consecutive days, enrolling a convenience sample of patients available on the respective days.*

5. In the description of the sample size calculation, they indicate that they have included 375 participants, but in reality, a portion were for the pilot study and 255 participants were actually used to measure metric properties.

*We thank the reviewer for alerting us to this misleading wording and we have rephrased the paragraph as follows:*

*“There is no consistent recommendation regarding sample size for the development and the evaluation of a questionnaire. A “rule of thumb” suggests at least 10 participants for each scale item [27,28].* *This would result in 160 participants for the 16-item version 1, or 130 participants for the 13-item version 3 of the questionnaire. We did not a priori decide on the exact number of items to be included in the questionnaire. Instead, we planned to conduct a pilot phase with at least 100 patients to optimize the development of the questionnaire. For the final analysis, we aimed to include at least 200 participants. Since we expected a drop-out rate of 20%, we opted for the overall inclusion of 375 patients.”*

*Revised manuscript with highlighted changes, p.7*

6. In the table 3 where they indicate the change in each item, it would be good to include the content of the item to assess the reason for the effect size.

*Thank you for this advice. We have added the item description to Table 3, p. 13.*

7. They have also calculated Cronbach's alpha for the total instrument. An important part of the validation of an instrument is to assess its factor structure and to check the unidimensionality. I do not know if the authors initiated any analysis in this regard although they did not mention it in the article.

*Indeed, we have performed confirmatory and exploratory factor analyses. The exploratory factor analysis showed the presence of at least four factors (based on the p-value of the chi-squared test which was smaller than 5% for three factors and 0.112 for four factors). Those factors were not congruent with the questionnaire from which we adapted the items which had two dimensions, a physical and a mental dimension. Since our questionnaire is based on a validated questionnaire with two dimensions, we additionally performed confirmatory factor analysis, checking whether our adapted questionnaire shares the same dimensions. The confirmatory factor analysis showed a root mean squared error of approximation of 0.085 (90% CI: 0.07 – 0.10), which does not show a good fit. In the same line, the other indices show a rather unsatisfactory fit. Also, removing some of the items with a small loading did not improve the result. Since the results of the factor analyses do not add to the psychometric robustness of the QoR-PACU, but rather point to the necessity of further modifications of the instrument, we did not report these results but instead aim to continue improving the questionnaire.*

*If requested by the editor or the reviewer, we will be happy to include the results of the factor analyses in the manuscript or as supplementary information.*

8. As a limitation, it should be pointed out that all the patients underwent radical prostatectomy surgery. This obviously implies that women have not been included, but also that the mean age of the sample will be mostly older adults.

*We strongly agree with the reviewer. The fact that we included solely male patients of similar age and ethnicity, scheduled for the same surgical procedure, is one of the main limitations of our study.*

*We have mentioned this limitation in the manuscript:*

*“This validation study was performed at the PACU of a prostate cancer clinic. All surgical procedures and perioperative care at our prostate cancer center are highly standardized. Although allowing for excellent comparability between participants, generalizability is limited. We included solely male patients scheduled for radical prostatectomy. Results from previous studies suggest that gender aspects have an impact on postoperative QoR and speed of recovery [2,42–44]. Overall, female patients tend to have lower QoR and longer PACU stay [2,42,44]. Morevover, pain intensity, nausea, and vomiting after surgery are more frequently reported by female patients [42,43]. Gender aspects may be of high importance in individualized perioperative care and postoperative recovery.*

*[…]*

*Future studies should evaluate the psychometric properties of the QoR-PACU in a more heterogenous patient population, including female and gender diverse patients, as well as a greater variety of patient-related and procedure-related risk factors.”*

*We aim to conduct a follow-up study which will include a more heterogenous study cohort including female as well as gender-diverse patients, different age groups and a variety of surgical procedures.*

*See revised article with highlighted changes, p.18-19*

9. Another limitation is that the patients were included in the development of the instrument but only for the pilot study or the final interviews. It is highly recommended to include patients and professionals in all phases of the development process, for example, through focus groups.

*We agree with the reviewer, that it would be commendable to include patients in all phases of development, including the very early stages. We did not develop the QoR-PACU from scratch. Instead, we selected items from the QoR-40. At this stage of development, only professionals (experienced anesthesiologists) were involved.*

*For the follow-up study we will modify the QoR-PACU. The modification will comprise suggestions from anesthesiologists, PACU nurses and patients.*

*We have modified the conclusion as follows:*

*“This preliminary validation study presents the development of a questionnaire to assess self-reported QoR after surgery in the PACU. We found high acceptability and feasibility, good validity, and adequate responsiveness. Against our hypothesis, we did not find high internal consistency. Based on these findings, the QoR-PACU should be modified. The modification process should consider suggestions from healthcare professionals and patients. Future psychometric evaluation should include a more heterogeneous patient cohort including female and gender-diverse patients with varying degrees of perioperative risk.”*

*See revised article with highlighted changes, p.19-20*

10. Although the article presents some preliminary analyses for the validation of the instrument, I believe that if the authors are able to address some comments and to emphasize the limitations by explaining that it is a preliminary validation study, the article is of interest.

*We thank the reviewer for this appraisal of our study. We have specified the term “preliminary validation study” in the conclusion (see comment above). To address the nature of the study more precisely as expressed by the reviewer, we suggest to edit the title of our manuscript into “Psychometric evaluation of a quality of recovery score for the postanesthesia care unit – a preliminary validation study*

*See revised article with highlighted changes, p.19-20*

***References***

1. Myles PS, Grocott MPW, Boney O, Moonesinghe SR, Group C-S. Standardizing end points in perioperative trials: towards a core and extended outcome set. Br J Anaesth. 2016;116: 586–589. doi:10.1093/bja/aew066

2. Demumieux F, Ludes P-O, Diemunsch P, Bennett-Guerrero E, Lujic M, Lefebvre F, et al. Validation of the translated Quality of Recovery-15 questionnaire in a French-speaking population. Br J Anaesth. 2020;124: 761–767. doi:10.1016/j.bja.2020.03.011

3. Lyckner S, Böregård I-L, Zetterlund E-L, Chew MS. Validation of the Swedish version of Quality of Recovery score -15: a multicentre, cohort study. Acta Anaesthesiol Scand. 2018;62: 893–902. doi:10.1111/aas.13086

4. Stark PA, Myles PS, Burke JA. Development and Psychometric Evaluation of a Postoperative Quality of Recovery Score. Anesthesiology. 2013;118: 1332–1340. doi:10.1097/aln.0b013e318289b84b
